# Application of MLST and Pilus Gene Sequence Comparisons to Investigate the Population Structures of *Actinomyces naeslundii* and *Actinomyces oris*

**DOI:** 10.1371/journal.pone.0021430

**Published:** 2011-06-30

**Authors:** Uta Henssge, Thuy Do, Steven C. Gilbert, Steven Cox, Douglas Clark, Claes Wickström, A. J. M. Ligtenberg, David R. Radford, David Beighton

**Affiliations:** 1 Department of Microbiology, The Henry Wellcome Laboratories for Microbiology and Salivary Research, King's College London Dental Institute, London, United Kingdom; 2 Department of Oral Biology, Faculty of Odontology, Malmö University, Malmö, Sweden; 3 Department of Oral Biochemistry, ACTA, Amsterdam, The Netherlands; University of Edinburgh, United Kingdom

## Abstract

*Actinomyces naeslundii* and *Actinomyces oris* are members of the oral biofilm. Their identification using 16S rRNA sequencing is problematic and better achieved by comparison of *metG* partial sequences. *A. oris* is more abundant and more frequently isolated than *A. naeslundii*. We used a multi-locus sequence typing approach to investigate the genotypic diversity of these species and assigned *A. naeslundii* (n = 37) and *A. oris* (n = 68) isolates to 32 and 68 sequence types (ST), respectively. Neighbor-joining and ClonalFrame dendrograms derived from the concatenated partial sequences of 7 house-keeping genes identified at least 4 significant subclusters within *A. oris* and 3 within *A. naeslundii*. The strain collection we had investigated was an under-representation of the total population since at least 3 STs composed of single strains may represent discrete clusters of strains not well represented in the collection. The integrity of these sub-clusters was supported by the sequence analysis of *fimP* and *fimA*, genes coding for the type 1 and 2 fimbriae, respectively. An *A. naeslundii* subcluster was identified with both *fimA* and *fimP* genes and these strains were able to bind to MUC7 and statherin while all other *A. naeslundii* strains possessed only *fimA* and did not bind to statherin. An *A. oris* subcluster harboured a *fimA* gene similar to that of *Actinomyces odontolyticus* but no detectable *fimP* failed to bind significantly to either MUC7 or statherin. These data are evidence of extensive genotypic and phenotypic diversity within the species *A. oris* and *A. naeslundii* but the status of the subclusters identified here will require genome comparisons before their phylogenic position can be unequivocally established.

## Introduction

*Actinomyces naeslundii* and *Actinomyces oris* are part of the commensal oral microbiota [1]–[3], may play a role in pathogenesis of caries [1] and have been isolated from extra-oral infections [4]. The identification of these species has proven difficult over the years since no reliable phenotypic characteristics are known to distinguish between either species [5] though for a period catalase production was used to distinguish between *A. naeslundii* and isolates then described as human *Actinomyces viscosus* [6]. Many of these difficulties were resolved by the extensive genetic studies reported by Johnson and colleagues [5] in which *A. naeslundii* genospecies 1, 2 and WVA 963, on the basis of DNA-DNA hybridization data, were described. Formal species descriptions were not given as they were unable to distinguish between the genospecies with phenotypic tests. However, genotype specific antisera were developed [7] which permitted the identification of *A. naeslundii* genospecies 1 and 2 from samples of dental plaque and infected dentine associated with dental caries [1]. Subsequent identification of isolates as members of genospecies 1 or 2 have relied on genospecies-specific antisera [7] which has enabled the distribution and phenotypic properties of the two genospecies to be compared. Thus *A. naeslundii* genospecies 2 isolates were demonstrated to bind to N-acetyl-β-D-galactosamine and acidic proline-rich proteins and to exhibit an N-acetyl-β-D-galactosamine binding specificity signified by N-acetyl-β-D-galactosamine-inhibitable coaggregation with the specified streptococcal strains. *A. naeslundii* genospecies 1 also bound to N-acetyl-β-D-galactosamine, but commonly not to acidic proline-rich proteins and possessed another N-acetyl-β-D-galactosamine binding specificity to a different set of streptococcal isolates [8]. However, the hemagglutination patterns of strains ascribed to genospecies 1 and 2 were not uniform indicating phenotypic heterogeneity of the surface properties within these taxa. Sequence analysis of the *fimA* gene, coding for the type 2 fimbriae, from isolates of the two genospecies indicated that there was greater similarity in sequence within genotypes than between genotypes. However, diversity of *fimA* sequences within each genotype was apparent and strains LY7 and P-5-N clearly harboured *fimA* genes with sequences different to those of the other three studied in each genotype although the *fimP* gene from 5 *A. naeslundii* genospecies 2 isolates exhibited ≥98 sequence homology. Members of these two genospecies also exhibited different patterns of interaction with other bacteria, red blood cells and salivary proteins [9]. These two genospecies are early colonisers of tooth surfaces but *A. naeslundii* genospecies 2 is isolated from the mouth more frequently and in greater numbers than *A. naeslundii* genospecies 1 [5], [10].

We have recently reported the descriptions of *Actinomyces naeslundii* (previously *Actinomyces naeslundii* genospecies 1), *Actinomyces oris* (previously *Actinomyces naeslundii* genospecies 2) and *Actinomyces johnsonii* (previously *Actinomyces naeslundii* genospecies WVA963) by means of sequence analysis of housekeeping genes [2]. We found that some isolates identified as genospecies 1 by Hallberg et al [8], [10] on the basis of their reactions with genospecies-specific antisera [7] were *A. oris* and that the sequenced actinomyces strain “*A. naeslundii*” MG-1 is a member of the species *A. oris* [2].

Previous studies have also reported on the intra- and inter-species diversities of *A. naeslundii* and *A. oris*, as *A. naeslundii* genospecies 1 and 2 using DNA fingerprinting [11], ribotyping [5], [12], amplified 16S ribosomal DNA restriction analysis (ARDRA) [4], [13], DNA probes [14], sequence analysis of *fimA* genes [9] and REP-PCR amplicons analysis [10]. All these studies have demonstrated extensive heterogeneity within each taxon. In this report we have applied a multilocus sequencing typing (MLST) approach [15] to investigate the diversity of the two species and used analysis of partial *fimA* and *fimP* gene sequences to validate the diversity apparent in the MLST analysis.

## Materials and Methods

### Bacterial strains and growth conditions

The partial sequences of 16S rRNA genes, selected house-keeping genes and genes for fimbrial proteins, fimA and fimP, were determined for strains in a collection of A. naeslundii, A. oris, A. johnsonii (Table S1). Additional gene sequence data were derived from the genomic data of “A. naeslundii” strain MG-1 (http://cmr.jcvi.org/tigr-scripts/CMR/GenomePage.cgi?org=gan). Isolates were stored at −80°C in cryo-preservative fluid. Bacteria were subcultured twice on fastidious anaerobe agar (FAA; Lab M Ltd.) supplemented with 5% defibrinated horse blood and cultivated anaerobically at 37°C for 24 to 48 h.

DNA was extracted using Proteinase K as described previously [16]. Briefly, cells were washed once in 2 M NaCl, centrifuged and resuspended in 50 µl TE buffer containing 0.5% Tween®20 (Tris-EDTA buffer, pH 8.0). Proteinase K solution (10 mg/ml stock solution) was added to a final concentration of 200 µg/ml. The tubes were incubated at 55°C for 2 h and subsequently heated at 95°C for 5 min. The tubes were centrifuged at 13,000 rpm for 1 min, the supernatants were transferred into new microcentrifuge tubes and the DNA extracts were stored at −20°C.

Partial 16S rRNA sequences (GQ421308–GQ421320 and JF776381–JF776392), approximately 1390 bp in length, of selected strains were determined with the universal primers 9f and 1512r for amplification and 9f, 519r, 357f, 1100r and 1492r for sequencing [17]. Partial sequences of the genes atpA [ATP synthase F1, alpha subunit, ANA_0169], metG [methionyl-tRNA synthetase, ANA_1898], rpoB [DNA-directed RNA polymerase, beta subunit, ANA_1497], pgi [glucose-6-phosphate isomerase, ANA_0727], gltA [citrate synthase I, ANA_1674] and gyrA [DNA gyrase, A subunit, ANA_2224] have been previously reported [2] (EU603149–EU603264 (pgi), EU620779–EU620894 (atpA), EU620895–EU621010, (gltA), EU621011–EU621126 (gyrA), EU621127–EU621242, (metG) and EU621243–EU621358 (rpoB))Partial sequences of pheS [phenylalanyl-tRNA synthetase, alpha subunit, ANA_1034] (GQ354571–GQ354683) were also determined using combinations of PheS-F (GACGAGGACGGCATCAAT, CATCGCCAAGGCTCTGA, TCGGCACCCTGGACAA and CTGGACAAGGCGGACAA) and PheS-R (CTGGGAGAAGCGGATGT; GCCGAACTGCTGGAGAA, ACCGCCCTTCTTCTGG, GAACCACAGGTCCATCT and CGAAGCCCGTGTAGACCT) primers to amplify a portion of the gene and PheS-sqF (CGCCGAGGAGGAGAT) and PheS-sqR (GCTGGGCTCGGTGAA) to sequence, in both directions, an internal fragment of the amplicon.

The PCR reactions were performed either in 0.2 ml microcentrifuge tubes or in non-skirted 96-well PCR plates (Thermo Scientific, UK) in a total volume of 15 µl. The reaction mix contained 1 µl DNA template, 0.2 µM of each primer (MWG), 0.5 mM MgCl_2_ and Reddy-Mix (Thermo Scientific, UK). Due to noticeable sequence variability and recurring high G+C mol% regions in pheS it was necessary to amplify and sequence the gene using multiple forward and reverse primers. When more than five primers were used in a PCR reaction the concentration of each primer was reduced to 0.16 µM. The PCR conditions for all amplification reactions were as follows: initial denaturation at 94°C for 10 min; 30 cycles at 94°C for 45 s, 53°C for 35 s, and 72°C for 75 s; and final extension at 72°C for 10 min. PCR products were cleaned adding 4 units Exonuclease I (Fermentas, Canada) and 1 unit Shrimp Alkaline Phosphatase (Thermo Scientific, UK) to each reaction which were then incubated at 37°C for 45 min and subsequently heated at 80°C for 15 min to inactivate the enzymes. The DNA fragments were sequenced using an ABI Prism cycle sequencing kit (BigDye terminator cycle sequencing kit with AmpliTaq DNA polymerase FS).

### *fimA* and *fimP* gene sequencing

Type-2 fimbriae *fimA* [ANA_0024 fimbrial structural subunit] and type-1 fimbriae *fimP* [ANA_2510 Type-1 fimbrial major subunit precursor] of strain MG-1 were used as the basis for the design of primers to amplify these genes. The partial *fimA* fragment analyzed equates to positions 739–1381 (659 bp) in the *A. oris* MG-1 gene and is located between the conserved regions of pilin motif and E box (JF825156–JF825240 (*fimA*) and JF825241–JF825307 (*fimP*)). The partial *fimP* fragment covered the positions 571–1234 in strain *A. oris* MG1 and was also located between the pilin motif and E box. It was necessary to use sets of primers (Table 1) in the initial amplification reaction due to the sequence diversity of the two pilus genes. For the PCRs the reaction mix contained 1 µl DNA template, 0.2 µM of each primer (MWG), 0.5 mM MgCl_2_ and Reddy-Mix (Thermo Scientific). The Reddy-Mix included 1.5 mM MgCl_2_, 0.2 mM each dNTP, 20 mM (NH_4_)_2_SO_4_, 75 mM Tris-HCl (pH 8.8 at 25°C), 0.01% (v/v) Tween®20 and Thermoprime Plus DNA Polymerase (0.025 units/µl) in a total volume of 15 µl. A multi-primer approach was utilized in which usually four primers were included, but up to six primers were included in some reactions. DNA amplification was carried out using the following programme: 94°C for 10 min, 10 cycles of 94°C for 45 sec, 56°C for 35 sec and 72°C for 75 sec followed by 20 cycles of 94°C for 45 sec, 53°C for 35 sec and 72°C for 75 sec and 72°C for 7 min for the final elongation. The amplicons were sequenced as described above.

**Table 1:** Primers used for the amplification and sequencing of the *fimA* and *fimP* genes.

PCRtarget	Primers	Primary primer sequence sets(5′-3′)	Sequencing primers(5′-3′)	bp
*fimA*	fimA-F(forward)	1.CCAAGCCCTTCGTGGT2.CATCCACAAGCACCTCA3.GGYRACATCGTCCAGAAG4.MMTGGMTCTACGAYGTCM5.GGGCGTCGGGCTGC6.CRCAGCCGGTGTCCTC	MACGTCTACCCYAAGAAC	659
	fimA-R(reverse)	1.CGGAACCGACTGCTTG2.CBGGWGCCTTGGWCTCA3.ACCGGTCAGGGGCAG4.GCGGTCAGGATGAGCAT5.GACGGCGATCATCAGCAG6.CBGGWGCCTTGGTCTC	GCCTTGGTCTCVACCAG	
*fimP*	fimP-F	1.GTGCGARCAGACCGACA2.CCKACGASGGCTGGAARAC3.CCCGCTGACCCGAAC4.TCACCATCACCAAGCTGAAC	CCACGTCTACCCCAAGAA	679
	fimP-R	1.SACGAGGCAGTAGTAGTCMT2.GCTTGTTGGCGTAGGC3.TCAGCTGGTCCTTCTTC4.AGCCAGGACCCGGAAG5.TTCTTCTCGGCCTTCTCAG6.CGCAGGTAGTTGATCTCCA	GGGGGCCTTGGTCTC	

### Analysis of house-keeping gene data and ST assignment

Chromatograms of forward and reverse sequences of *pheS* were analysed, trimmed and aligned using BioEdit [18]. The sequence data of the *A. oris* and *A. naeslundii* for each locus were analysed and unique allele sequences were given continuous numbers and every unique allelic profile was given a unique sequence type (ST) [15]. STs were assigned to all *A. oris* and *A. naeslundii* isolates such that STs 1–68 were *A. oris* and STs 69–100 were assigned to the *A. naeslundii* strains. A neighbor-joining tree was constructed in MEGA4 [19] using the concatenated sequences of each ST and bootstrap values calculated based on analysis of 1000 resampled datasets.

Further phylogenetic analysis of the sequence data of all *A. oris* and *A. naeslundii* STs was performed using ClonalFrame [20] a technique widely used to assess the evolutionary relationships between strains of the same and closely related bacterial species as it enables bacterial recombination to be taken into consideration when constructing phylogenetic history. The threshold for the consensus tree was set to 0.50 and 0.95. Six ClonalFrame runs were conducted using the default settings, 50,000 iterations which were discarded, followed by 50,000 iterations of which every 100^th^ generation was sampled. Therefore, 501 trees per run were calculated and the data of the six runs were combined and a consensus phylogenetic tree was drawn in MEGA4 [19].

### 16S rRNA sequence analysis

Partial 16S rRNA gene sequences of selected *A. oris* and *A. naeslundii* isolates identified as outliers to the main species groups were determined. A Neighbor-joining tree was constructed in MEGA4 and bootstrap values calculated based on analysis of 1000 resampled datasets. The tree included the sequences determined here and the sequences for the type strains of *A. viscosus* (X82453), *A. naeslundii* (X81062), *A. johnsonii* (AB545933.1) and *A. oris* (GQ421308).

### The fimA and fimP sequences

These sequences were trimmed and edited using BioEdit. The derived sequences were aligned with *fimA* gene of four *A. naeslundii* genospecies 2 strains [*A. oris*] (DQ425102, DQ425099, DQ425101 and AF019629) and three *A. naeslundii* genospecies 1 strains [*A. naeslundii*] (DQ425097, DQ425098 and DQ425100). The *fimA* sequence of *A. odontolyticus* strain PK984 (DQ425103) was also included in these analyses as were the *fimP* gene sequences of three *A. naeslundii* genospecies 2 strains [*A. oris*] (AF106053, AF107019 and AF107020), human ‘*A. viscosus*’ ATCC19249 (AF106034) and the *fimA* and *fimP* of *A. oris* MG-1. Neighbor-joining trees were constructed in MEGA4 for the *fimA* and *fimP* partial gene sequences and bootstrap values calculated based on analysis of 1000 resampled datasets.

### Binding assays

The strains selected for these assays were cultured anaerobically at 37°C on Fastidious Anaerobe Agar (LabM, Bury, UK) supplemented with 5% (v/v) defibrinated horse blood and single colonies transferred to brain-heart infusion broth and grown anaerobically for 2 days. These cultures were used to inoculate Todd Hewett Broth (Oxoid) and grown anaerobically for 2 days. The cells were harvested by centrifugation (2000 g for 10 min), washed and resuspended in 1–3 ml saliva buffer (2 mM KH_2_PO_4_/K_2_HPO_4_, 50 mM KCL, 1 mM CaCl_2_, 0.1 mM MgCl_2_) containing 0.02% NaN_3_ and stored at 4°C prior to use.

Parotid saliva was obtained from a volunteer using a sterilized Lashley suction cup placed over the opening of a Stenson's duct; parotid saliva flow was stimulated by sucking on a sugar-free lemon sweet (Simkins, Sheffield, UK). Ethical permission for the collection of human parotid saliva was obtained from the Ethics Committee of Guy's and St Thomas Hospital Foundation Trust. Written consent for the collection of the saliva sample was obtained.

A proline-rich protein (PRP)-enriched fraction was obtained from parotid saliva by boiling to remove amylase [21], selective adsorption of statherin with hydroxyapatite [22] and precipitation of histatins with ZnCl_2_ [23]. The predominance of PRPs in the resulting preparation was confirmed by SDS-PAGE and Coomassie Blue staining. Gel and sol phases of purified human MUC5B (high molecular weight salivary mucin, MG1) and an enriched sol fraction of MUC7 (low molecular weight salivary mucin, MG2) were prepared as previously described [24], [25]. Protein concentrations were measured using a Pierce BCA kit with a BSA standard (Thermo Scientific). In the case of mucins, the figures were adjusted to take into account their protein percentages [26] for total concentrations.

Saliva and salivary proteins were coated on to MaxiSorb plates (Thermo Scientific) by overnight incubation of dilutions in buffer at 100 µl per well. The PRP fraction were used respectively at concentrations of 4.0, 0.25 and 0.25 µg/ml in 0.1 M NaHCO_3_/NaCO_3_ buffer, pH 9.6, at 4°C. The MUC5B gel and sol phases and the MUC 7 fraction were used at concentrations of 110, 40 and 1.0 µg/ml respectively in phosphate-buffered saline (PBS) at room temperature. All plates were washed 4× with PBS containing 0.1% Tween 20 (PBS-T). Microwell plates coated with 2.5 µg/ml of human statherin were prepared as previously described [27] except that the statherin was coated by overnight incubation in sodium carbonate buffer (pH 9.6).

For the attachment experiments plates the PRP fraction were prepared in saliva buffer with and without 25 mM lactose [28], [29]. With mucin-coated plates the Actinomyces were resuspended in PBS-T at a concentration of approximately 5×10^8^ per ml. The latter buffer was also used for subsequent washing steps with all these types of plate. For the statherin plates, Tris-buffered saline (TBS) containing 1 mM CaCl_2_ and 0.1% Tween was used to resuspend bacteria and subsequent washing steps. Duplicate aliquots (100 µl) of the bacterial suspensions were used, the plates were incubated at 37°C for 60 min to allow bacterial attachment, the supernatants decanted and the plates washed 4× with buffer. Attached bacteria were stained [30] by incubation at 37°C for 30 min with 100 µl of a 5 µM solution of SYTO 13 (Invitrogen, Paisley, UK). The dye was removed and the fluorescence determined (Labsystems Fluoroskan, Thermo Scientific). To standardize the attachment experiments, initial fluorescence measurements were made with serial dilutions in 100 µl of saliva in the wells of microplates. The bacteria were spun down (2750 g for 30 min at 15°C) and the cells stained with SYTO 13. For subsequent experiments, bacteria were diluted to give a calculated fluorescence of 100 arbitrary units if complete attachment were achieved; in all assays background fluorescence, measured in the absence of labeled bacteria, was subtracted from test fluorescence values. For each type of plate coating, attachment measurements were made with 3 separate cultures of each strain used.

## Results

**Figure 1:**
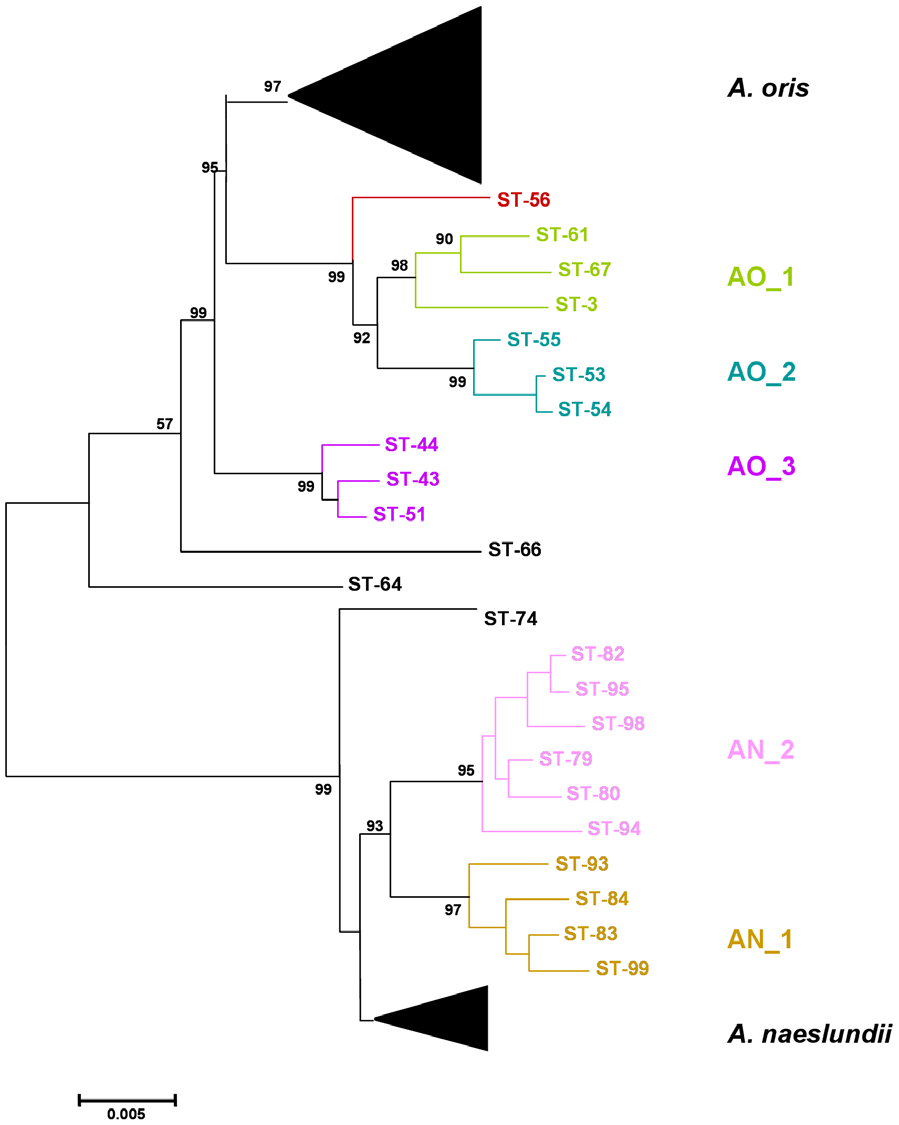
Neighbor-Joining tree of concatenated sequences of the 7 house-keeping genes of *A. naeslundii* and *A. oris*. Bar is 0.005 substitutions per site.

**Figure 2:**
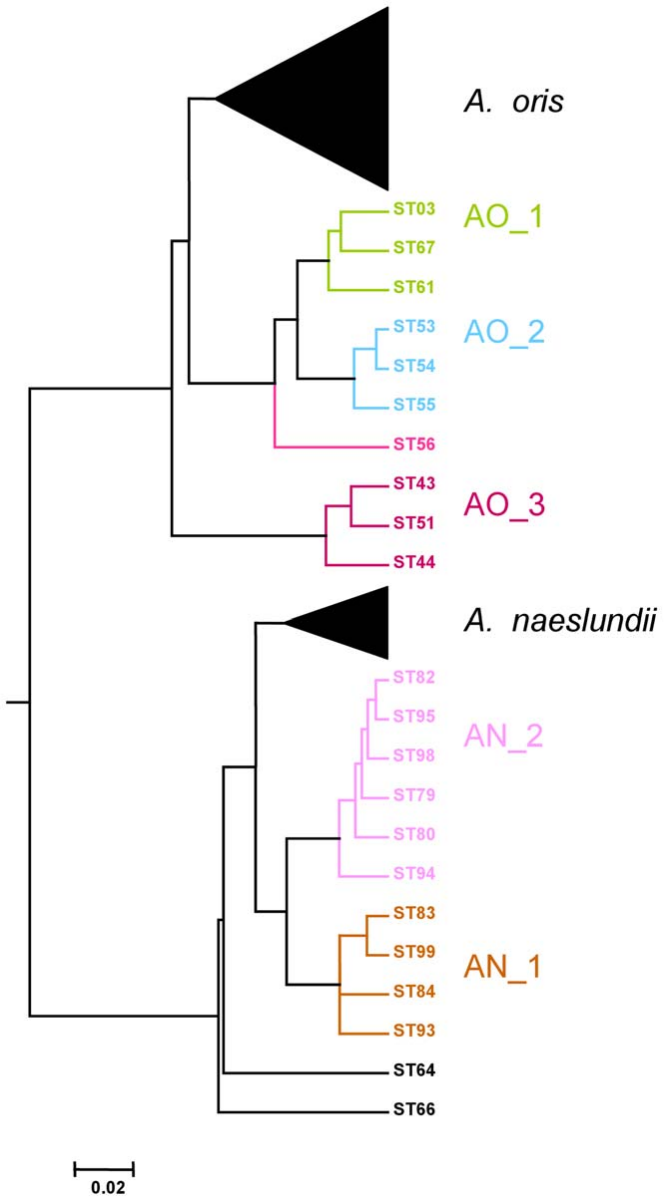
50% majority-rule consensus tree derived using ClonalFrame. Six ClonalFrame runs were conducted using the default settings, the initial 50,000 iterations were discarded and the next 50,000 iterations were sampled at every 100^th^ generation was sampled. Therefore, 501 trees per run were calculated and the data of the six runs were combined and the consensus tree was drawn in MEGA4 [19]. Scale is coalescent units.

The 37 *A. naeslundii* isolates were assigned to 32 unique allelic profiles (STs) while all 68 *A. oris* isolates yielded unique STs. A neighbor-Joining tree of the concatenated sequences from both species is shown in Fig. 1. The majority of *A. naeslundii* STs formed a single cluster which contained the *A. naeslundii* type strain and two additional clusters, AN_1 of 4 STs and AN_2 of 7 STs, were evident supported by bootstrap values of >90% with ST-74 on the periphery of the cluster. A greater level of diversity was found with the *A. oris* STs in which the majority of STs formed a single cluster which contained the *A. oris* type strain and addition discrete clusters labeled AO_1 to AO_3 and with ST-56, ST-66 and ST-64 on the periphery of the *A. oris* clusters. Analysis of the ST data using ClonalFrame produced similar relationships between the *A. oris* STs (Fig. 2). AO_1 to AO_3 identified but STs 66 and 64, were apparently more closely related to *A. naeslundii* than to *A. oris* and ST-56 more closely related to *A. oris* but still peripheral. The clusters, AN_1 and AN_2, were discrete but ST-74 was within the *A. naeslundii* cluster containing the type strain of the species.

**Figure 3:**
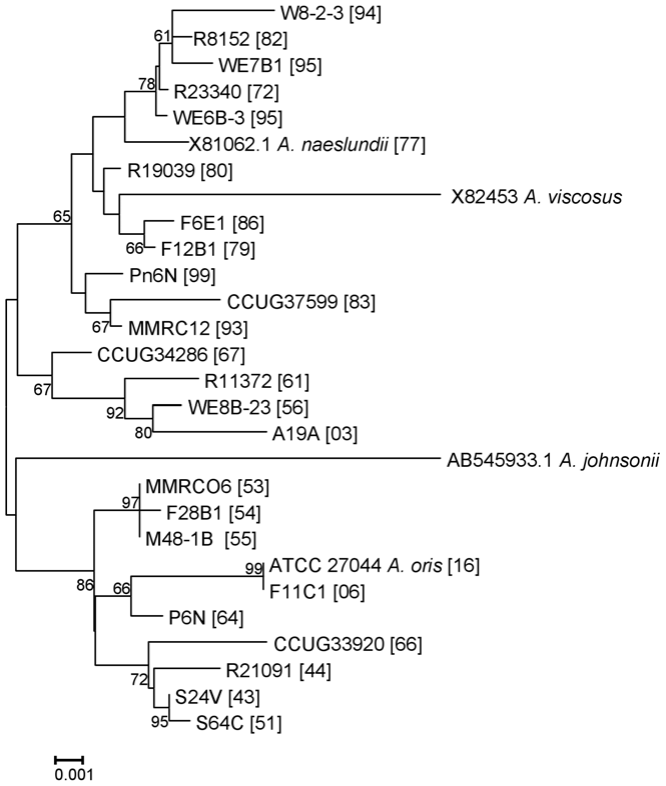
Neighbor-Joining tree of partial 16S rRNA gene sequences. Approximately 1400 bp sequences of 16S rRNA genes selected *A. naeslundii* and *A. oris* strains indicated by strain name and [sequence type] were determined. Sequences of *A. naeslundii* (X81062.1; ST77), *A. viscosus* (X82453), *A. johnsonii* (AB545933.1) and *A. oris* (GQ421308; ST16) type strains were included for comparative purposes. STs 1–68 are *A. oris* and 69–100 are *A. naeslundii*. Only bootstrap values of >60 are shown for clarity. Bar is 0.001 substitutions per site.

**Figure 4:**
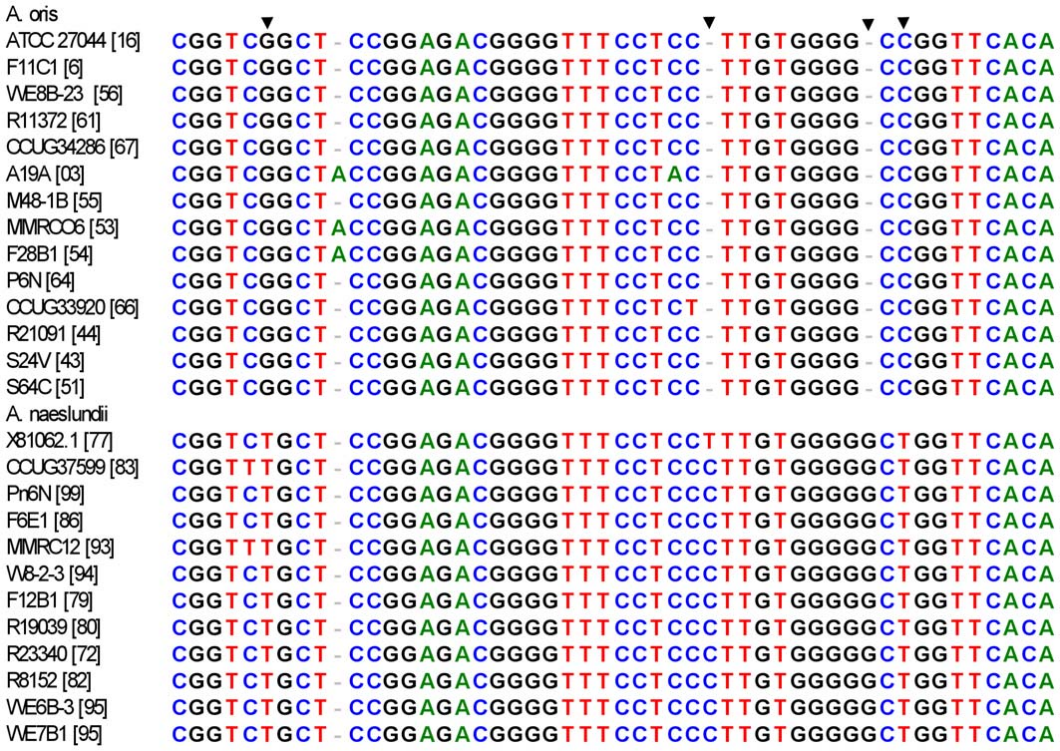
16S rRNA signature differentiating *A. naeslundii* and *A. oris* 16S rRNA sequences. 16S rRNA sequences were aligned using BioEdit [18] and visually compared to detect sequence signatures that differentiated bewteen the species. Figure lists strain names and [ST]. The first base is base 1010 in 16S rRNA sequence of *A. naeslundii* NCTC 10301 (X81062). Signature bases are marked with arrowheads.

A neighbor-joining tree based on alignment of the 16S rRNA sequences of the selected STs, those not within the major groupings of the two species, is shown in Fig. 3. Overall the strains originally assigned to either *A. oris* or *A. naeslundii* formed two clusters but the bootstrap value supporting this division was not high. However, all the strains in the clusters AO_1 to AO_3 and STs 56, 66 and 64 were in a cluster with the type strain of *A. oris*. All strains in the clusters AN_1 and AN_2 and ST 74 were in a cluster that contained the type strain of *A. naeslundii* but also the type strain of *A. viscosus*. When the partial 16S rRNA sequences of the *A. oris* and *A. naeslundii* isolates were inspected a region that enabled differentiation between these species was identified (Fig. 4). Within the *A. naeslundii* cluster there were no sub-clusters that were congruent with the clusters observed in Fig. 1, however, some degree of congruence was apparent within the *A. oris* strains. Thus the strains in AN_1 formed a loose cluster in Fig. 3 which also included ST-56 while clusterAN_2 was discrete in both analyses as was AN_3 but which also included ST-66. ST-64 was more closely related to *A. oris* ATCC 27044, the type strain for the species.

The 16S rRNA sequence confirmed the allocation of isolates to *A. oris* and *A. naeslundii* on the basis of house-keeping genes. However, analysis of the concatenated sequences of 7 house-keeping genes, by neighbor-joining trees and ClonalFrame analysis, suggested that there existed within both species phylogentically distinct clusters which might constitute subspecies and this was supported, at least within the *A. oris* by a comparison of the partial 16S rRNA sequences. To investigate this further, we determined partial sequences of *fimA* and *fimP* to determine if the same phylogenetic relationships could be demonstrated using these genes for extracellular proteins.

**Figure 5:**
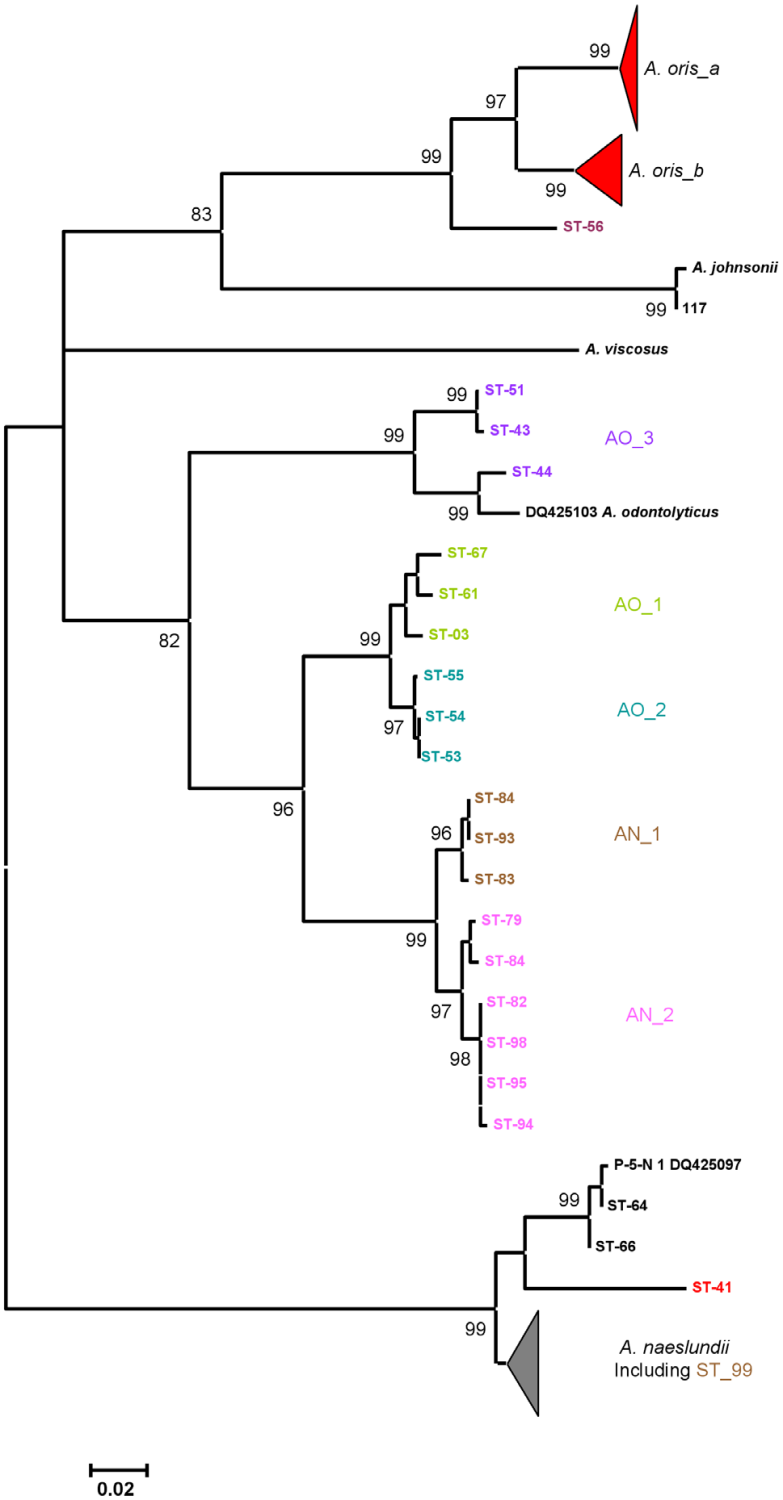
Neighbor-joining tree showing relationships between *fimA* sequences. Partial *fimA* sequences of *A. oris*, *A. naeslundii*, *A. johnsonii* and *A. viscosus* strains were determined, aligned using BioEdit [18] and a neighbour-joining tree was calculated and visualised using MEGA4. Strain 117 is an additional *A. johnsonii* strain, see Table S1.

The neighbor-joining tree based on partial *fimA* sequences of the *A. oris*, *A. naeslundii*, *A. johnsonii*, *A. viscosus* and *A. odontolyticus* are shown in Fig. 5. The major *A. oris* cluster identified in Fig. 1 was discrete but split into two subclusters. A.oris_a contained the sequence from the *A. oris* type strain and the sequences DQ425099 and DQ425102 while the A.oris_b contained the sequence from MG-1 and the sequence DQ425101. The fimA sequence from ST-56 was peripheral to these clusters. Strains in AO_1 and AO_2 were present in discrete clusters in the *fimA* tree while AO_3 was also present in a discrete cluster that contained the *A. odontolyticus fimA* sequence DQ425103. The remaining *A. oris* STs, 64 and 66, formed a cluster which included DQ425097 which was closely related to, but distinct from, the *fimA* sequences of *A. naeslundii*. The strains from clusters AN_1 and AN_2 also formed distinct clusters distinct from both the major *A. oris* and *A. naeslundii* clusters. ST-99, a member of the AN_1 cluster was now grouped within the major *A. naeslundii* cluster. The sequences from the *A. johnsonii* strains formed a discrete cluster and the sequence from the *A. viscosus* type strain was discrete and the sequence from ST-41 was on the periphery of the major *A. naeslundii* cluster.

**Figure 6:**
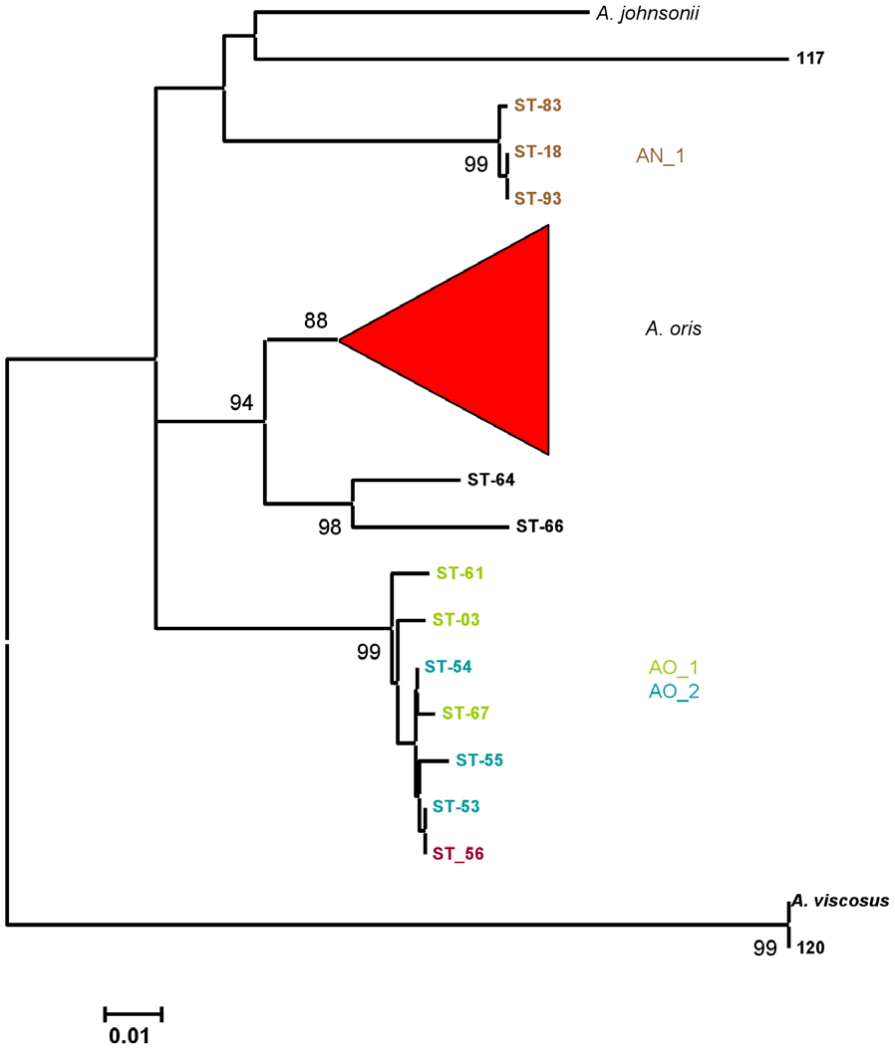
Neighbor-joining tree showing relationships between *fimP* sequences. Partial *fimA* sequences of *A. oris*, *A. naeslundii*, *A. johnsonii* and *A. viscosus* strains were determined, aligned using BioEdit [18] and a neighbour-joining tree was calculated and visualised using MEGA4. *fimP* sequence 120 is from *A. viscosus* ATCC19246 [AF106034]. Strain 117 is an additional *A. johnsonii*, see Table S1.

The neighbor-joining tree based on the partial *fimP* sequences is shown in Fig. 6. The *A. johnsonii* and *A. viscosus* strain sequences were discrete from those of *A. oris* and *A. naeslundii*. Gene *fimP* was only detected in 3 STs identified as *A. naeslundii* and these all corresponded to 3 of the 4 STs in AN_1. The other member of AN_1, ST-99, which also harbored a *fimA* different to that of the other members of AN_1 did not reveal *fimP*. A single cluster consisting of the STs in AO_1, AO_2 and ST-56 was evident, discrete from the major *A. oris* cluster. Strains in AO_3 did not harbor *fimP*. Two STs, 64 and 66, were discrete and on the periphery of the major *A. oris* cluster.

### Actinomyces binding to salivary proteins

**Figure 7:**
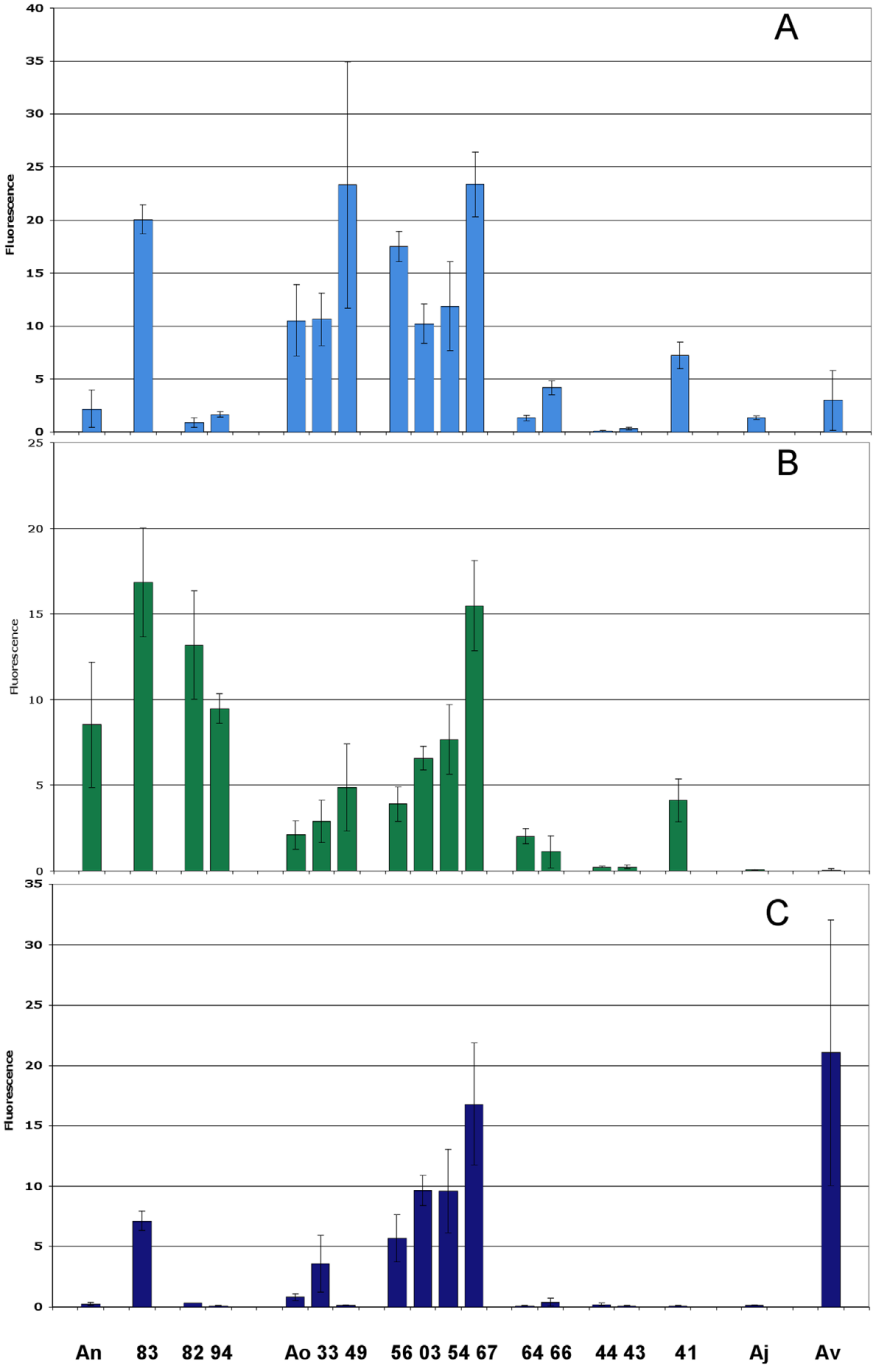
Relative attachment of actinomyces strains to salivary proteins. Bacteria were bound to salivary proteins and saliva preparations and labelled using the fluorescent stain SYTO 13. Bacterial cell binding was normalised such that maximum was 100 and equivalent to all added bacteria binding to substrate. The substrates were (A), a preparation of proline-rich proteins in the presence of lactose (B) MUC7 and (C) statherin. An is the *A. naeslundii* type strain [ST 77], Ao is the *A. oris* type strain [ST 16], Aj is the *A. johnsonii* type strain and Av is the *A. viscosus* type strain.

The *A.naeslundii* STs 83 (AN_1), 82 and 94 (AN_2) and the *A. naeslundii* type strain all bound to MUC7 (Fig. 7) but only ST-83 bound to a significant extent to both the PRP preparation and to statherin. *A. oris* strains ST-33 and 49 as well as the type strain bound to both MUC7 and the PRP preparation but only weakly to statherin. ST56, ST-3 (AN_1) and STs 54 and 67 (AN_2) exhibited high binding to MUC7, the PRP preparation and to statherin while STs 64 and 66 exhibited only weak binding to MUC7 and the PRP preparation. The two members of AO_3 examined (STs 44 and 43) exhibited very low binding to all salivary proteins. *A. oris* ST-41 exhibited binding only to MUC7 and the PRP preparation. The *A. johnsonii* type strains exhibited very low binding to all salivary proteins while the *A. viscosus* type strains exhibited high binding to statherin, no binding to MUC7 and low binding to the PRP preparation. The patterns of binding observed with MUC7 were also apparent with the MUC5B gel and sol preparations. The ability of the selected strains to bind to the salivary proteins, their cluster identity and the presence of *fimA* and *fimP* are summarized in Table 2.

**Table 2:** Summary of the ST binding characteristics.

	Cluster^1^	*fimA* 2	*fimP* 2	Binding to:		
				MUC7^3^	PRP^3^	Statherin^3^
*A. naeslundii*						
ST-77 [ATCC12104 = NCTC 10301]	Main	+	−	3+	+	+/−
ST-94	AN_2	+^a^	−	3+	+	+/−
ST-82	AN_2	+^a^	−	4+	+	+/−
ST-83	AN_1	+^b^	+	5+	4+	3+
*A. oris*						
ST-16 [ATCC27044]	Main	+	+^a^	2+	3+	+
ST-33	Main	+	+^a^	2+	3+	2+
ST-49	Main	+	+^a^	2+	5+	+/−
ST-56	Lone	+^a^	+^b^	2+	4+	3+
ST-03	AO_1	+^b^	+^b^	3+	3+	3+
ST-54	AO_2	+^c^	+^b^	3+	3+	3+
ST-67	AO_1	+^b^	+^b^	5+	5+	4+
ST-64	Lone	+^d^	+^c^	2+	1+	+/−
ST-66	Lone	+^d^	+^c^	+	2+	+/−
ST-44	AO_3	+^e^	+^a^	+/−	+/−	+/−
ST-43	AO_3	+^e^	+^a^	+/−	+/−	+/−
ST-41	Lone	+^f^	+^a^	2+	2+	+/−
*A. johnsonii*		+	+	+/−	+	+/−
*A. viscosus*		+	+	+/−	2+	5+

1 Clusters as identified in Figure 1.

2 Within each species the superscripts for *fimA* and *fimP* designate different partial *fimA* or *fimP* gene sequences.

3 Binding scores are an arbitrary range from − to 5+.

## Discussion

The taxonomy of the Actinomyces “viscosus-naeslundii” group has been the subject of much research in order to find valid and reproducible methods to differentiate between these species which were isolated from both humans and rodents. Phenotypic methods to discriminate between these species have proved elusive [5] although for a time *A. viscosus* was, incorrectly, differentiated from *A. naeslundii* on the basis of catalase production [6]. Serological methods were applied to the discrimination of these species [7] but it was not until the extensive taxonomic study of Johnson and colleagues [5] that these serological data could be understood in relation to a definite taxonomy. Thus by applying DNA-DNA homology it was shown that human strains of “viscosus-naeslundii” group were divided in three different species as the DNA homology between them was <70% [5]. The proposed classification was: *A. naeslundii* genospecies 1, *A. naeslundii* genospecies 2 and the separate *Actinomyces* serotype WVA 963. The animal strain was classified as *A. viscosus*. In that study, these four taxa shared between 26 to 44% DNA relatedness to each other. A slightly higher similarity was found between *A. viscosus* and *A. naeslundii* genospecies 1 (55%). Furthermore, it was shown that within *A. naeslundii* genospecies 2, strains from *A. naeslundii* serotypes II, NV and *A. viscosus* serotype II were closer related to each other (63 to 79%) than to *A. naeslundii* serotype III (51 to 62%). Subsequently, antisera were used in many studies [1], [10] to identify *A. naeslundii* from the human oral cavity as genospecies 1 or 2. 16S rRNA sequence analysis was widely used to identify many taxa but this approach was reported previously to be suitable for the discrimination of *A. oris* and *A. naeslundii*. However, our analysis here shows that there are limited but discrete sequence differences between these species and that these human species are separable but with a low bootstrap value. However, we [2] previously used a concatenated gene sequencing approach to discriminate between strains identified as *A. naeslundii* and were able to amend the description of *A. naeslundii* (genospecies 1) and described new species *A. oris* (genospecies 2) and *A. johnsonii* (WVA 963).

Here we describe some of the divergence present within *A. oris* and *A. naeslundii* using an MLST approach supplemented with an analysis of the partial sequences of *fimA* and *fimP* genes. Within both species discrete clusters, characterized by high bootstrap values, were identified and a significant proportion of the collection of each species was present in these discrete clusters. Examination of the Neighbor-joining or ClonalFrame dendrograms suggests that the strain collection we had investigated was an under-represented of the total population since, at least, STs 56, 64 and 66 appear to represent discrete clusters of strains not well represented in the collection.

Previously the *fimA* gene, the major subunit of type-2 fimbriae, was sequenced in four strains for each “*A. naeslundii* genospecies 1 and 2” revealing conserved regions as well as sequence differences [9]. The *fimP* gene, major subunit of type-1 fimbriae, was obtained from four strains of “*A. naeslundii* genospecies 2” and a human “*A. viscosus*” strain [31]. The *fimA* and *fimP* genes are distinct from the housekeeping genes used to construct the neighbor-joining tree. However, the relationships between the STs in the house-keeping gene tree and the *fimA* tree are very similar. Thus STs in AN_1 and AN_2 form discrete clusters in the fimA tree distinct from the main *A. naeslundii* cluster although ST-99 [AN_1] harbored a *fimA* gene indistinguishable from that of the majority of *A. naeslundii* strains. Similarly with the *A. oris* strains AO_1, AO_2 and AO_3 formed discrete clusters with *fimA* although AO_3 formed a cluster with the *A. odontolyticus* fimA sequence [DQ425103]. This suggests that this gene was acquired by horizontal gene transfer from *A. odontolyticus* while ST-99 appears to have acquired a *fimA* gene from a member of the main *A. naeslundii* cluster supporting our earlier observation of horizontal gene transfer between *A. oris* an *A. naeslundii* [32]. The two STs 64 and 66, which on the basis of the neighbor-joining tree were more similar to *A. oris* while the ClonalFrame analysis suggested they were more closely related to *A. naeslundii* formed a small cluster adjacent to the *A. naeslundii fimA* sequences which also included the sequence of P-5-N1 [DQ425097]. The sequence of *A. oris* ST-41 was closely related to that of *A. naeslundii*. The *A. johnsonii* and *A. viscosus* sequences were discrete while the sequence of ST-56, a lone ST in the neighbor-joining analysis, was also discrete but closely related to the *A. oris* cluster.

Type-1 fimbriae (*fimP*) were mainly found in “*A. naeslundii* genospecies 2” and may be necessary for early colonization of the tooth through binding to the acquired pellicle. They promote protein-protein binding to tooth-adsorbed salivary acidic proline-rich proteins (PRPs) and statherin [33]–[35]. Bacteria only bind to PRPs when the proteins are bound to a surface at which they undergo a conformational change [36]. Furthermore, different “*A. naeslundii* genospecies 2” strains exhibited varied binding to either PRPs or statherin [37]. Type-2 fimbriae (fimA) were identified in both *A. naeslundii* genospecies 1 and 2 [38]. This type of fimbriae binds to β-linked galactose and galactosamine structures [39] and thus facilitates binding to glycolipids and glycoproteins on surfaces of epithelial cells and bacteria [40], [41] as well as to the enamel pellicle [37]. Binding via the type-2 fimbriae is lactose inhibited. *fimP* is not present in the majority of *A. naeslundii* strains and here we only detected it in the three strains of AN_1. This sequence was distinct from all other *fimP* sequences. STs of AO_1 and AO_2 and ST-56 possessed highly similar *fimP* sequences, distinct from those of the main *A. oris* cluster, as did the two lone STs 64 and 66. Members of AO_3 were similar to those of the major group of *A. oris* strains. *A. viscosus* and *A. johnsonii* were distinct from all other sequences.

The binding properties of the selected strains overall mirrored the presence of the particular fimbriae genes. Thus, *A. naeslundii* STs 77, 94 and 82 adhered to well MUC7 while exhibiting only a low ability to bind to the PRP preparation or statherin. *A. naeslundii* ST-83, which possessed both *fimA* and *fimP* gene adhered well to all three salivary preparations. Although all *A. oris* STs possessed both *fimA* and *fimP* a number of different binding profiles were observed. Thus, members of the main *A. oris* cluster, AO_1 and AO_2 tended to adhere to all substrates, as did ST-56 while both AO_3 strains exhibited very low binding. The lone STs, 41, 64 and 66 exhibited overall low binding to the three saliva preparations as did *A. johnsonii* but *A. viscosus* exhibited high binding to statherin as has been previously reported.

In conclusion these data clearly demonstrate considerable population diversity within each of the species *A. oris* and *A. naeslundii* and show that there is a phenotypic basis to the clusters generated by the analysis of the concatenated sequences of 7 housekeeping genes. The population structure was congruent with that obtained by an analysis of the *fimA* and *fimP* partial gene sequences. It is inappropriate to attempt to assign names to these discrete clusters but these data provide a basis for further study to accurately plot the establishment and distribution of these organisms in the human mouth and perhaps to better understand their role in the health of the oral cavity. The phylogenic relationships between these clusters may be elucidated by the application of whole genome sequence comparison techniques [42].

## Supporting Information

Table S1 **Allelic profiles and sequence types of the** ***A. oris*** **and** ***A. naeslundii*** **strains.**
(DOC)
