# Supplementary material for: Application of MLST and Pilus Gene Sequence Comparisons to Investigate the Population Structures of Actinomyces naeslundii and Actinomyces oris
Source: PLoS One. 2011 Jun 30;6(6):e21430. doi: 10.1371/journal.pone.0021430 (PMC3127948; doi:10.1371/journal.pone.0021430)
Supplement: Table S1 — Allelic profiles and sequence types of the A. oris and A. naeslundii strains. (DOC) [file pone.0021430.s001.doc]

Table S1. Allelic profiles and sequence types of the *A. oris* and *A. naeslundii* strains.

| Strain name | *atpA* | *gltA* | *gyrA* | *metG* | *pgi* | *pheS* | *rpoB* | ST |
| --- | --- | --- | --- | --- | --- | --- | --- | --- |
| *A. oris* |  |  |  |  |  |  |  |  |
| A18A-3 | 1 | 1 | 1 | 1 | 1 | 1 | 1 | 1 |
| M47-1-1 | 1 | 32 | 8 | 30 | 8 | 6 | 9 | 2 |
| A19A-1 | 2 | 2 | 2 | 2 | 2 | 2 | 2 | 3 |
| A2A-1 | 3 | 3 | 3 | 3 | 3 | 3 | 3 | 4 |
| A3A-2 | 4 | 4 | 4 | 4 | 4 | 4 | 4 | 5 |
| F11C1 | 4 | 4 | 4 | 7 | 6 | 4 | 7 | 6 |
| F5A10 | 4 | 4 | 4 | 7 | 11 | 10 | 13 | 7 |
| WE10B-1 | 4 | 20 | 4 | 7 | 21 | 10 | 13 | 8 |
| MMRCO2-1 | 4 | 29 | 31 | 9 | 11 | 10 | 13 | 9 |
| P5K | 4 | 29 | 31 | 38 | 11 | 10 | 6 | 10 |
| A6A-1 | 5 | 5 | 5 | 5 | 4 | 5 | 5 | 11 |
| UN4B6c | 6 | 4 | 20 | 4 | 19 | 4 | 4 | 12 |
| F21A1 | 6 | 5 | 8 | 9 | 5 | 6 | 9 | 13 |
| A7A-1 | 6 | 6 | 6 | 6 | 5 | 6 | 6 | 14 |
| F24C1 | 6 | 6 | 10 | 11 | 8 | 6 | 9 | 15 |
| A. oris^T^ (ATCC27044) | 6 | 6 | 49 | 45 | 5 | 6 | 9 | 16 |
| F1a1 | 7 | 7 | 7 | 8 | 1 | 7 | 8 | 17 |
| F23A1 | 8 | 8 | 9 | 10 | 7 | 8 | 10 | 18 |
| F7j1 | 8 | 8 | 9 | 10 | 14 | 3 | 28 | 19 |
| F2A12 | 8 | 8 | 11 | 12 | 9 | 9 | 11 | 20 |
| R23277 | 8 | 8 | 43 | 39 | 7 | 8 | 10 | 21 |
| WE9A10-1 | 8 | 34 | 9 | 13 | 7 | 8 | 10 | 22 |
| F4D1 | 9 | 9 | 9 | 13 | 10 | 8 | 12 | 23 |
| S62B | 9 | 25 | 27 | 25 | 28 | 23 | 26 | 24 |
| U149-1 | 9 | 27 | 29 | 27 | 30 | 25 | 17 | 25 |
| M46-1-1 | 9 | 31 | 33 | 9 | 33 | 6 | 6 | 26 |
| R14746 | 9 | 39 | 11 | 7 | 36 | 34 | 32 | 27 |
| CCUG 34285 | 9 | 47 | 47 | 43 | 43 | 42 | 38 | 28 |
| S55N | 10 | 3 | 23 | 10 | 26 | 22 | 15 | 29 |
| G128C | 10 | 10 | 12 | 10 | 12 | 11 | 14 | 30 |
| G140C | 10 | 11 | 13 | 14 | 13 | 3 | 15 | 31 |
| S33A | 10 | 24 | 12 | 10 | 26 | 21 | 24 | 32 |
| G48B | 11 | 12 | 14 | 10 | 14 | 3 | 15 | 33 |
| R6630 | 11 | 37 | 38 | 10 | 14 | 3 | 15 | 34 |
| G53E | 12 | 13 | 15 | 15 | 15 | 12 | 10 | 35 |
| G54C | 13 | 14 | 16 | 16 | 16 | 13 | 16 | 36 |
| T22P-A1 | 14 | 15 | 17 | 14 | 17 | 14 | 17 | 37 |
| T5P-1 | 15 | 16 | 18 | 17 | 18 | 12 | 18 | 38 |
| T6P-2 | 16 | 17 | 19 | 18 | 17 | 15 | 19 | 39 |
| T8-1 | 17 | 18 | 13 | 14 | 13 | 16 | 15 | 40 |
| W11-1-1 | 18 | 19 | 21 | 19 | 20 | 17 | 20 | 41 |
| WE1Aa1 | 19 | 20 | 22 | 6 | 5 | 6 | 9 | 42 |
| S24V | 19 | 22 | 24 | 21 | 25 | 20 | 23 | 43 |
| R21091 | 19 | 40 | 40 | 36 | 38 | 35 | 23 | 44 |
| WE3Bc14 | 20 | 3 | 23 | 20 | 22 | 18 | 16 | 45 |
| WE4Bb1 | 21 | 12 | 12 | 10 | 23 | 19 | 21 | 46 |
| WE5Ba2 | 22 | 21 | 1 | 7 | 24 | 1 | 22 | 47 |
| S29C | 23 | 23 | 12 | 22 | 26 | 3 | 15 | 48 |
| S47B | 24 | 12 | 25 | 23 | 26 | 22 | 15 | 49 |
| S56D | 25 | 16 | 26 | 24 | 27 | 14 | 25 | 50 |
| S64C | 26 | 26 | 28 | 26 | 29 | 24 | 23 | 51 |
| MMRCO1-1 | 27 | 28 | 30 | 28 | 31 | 26 | 16 | 52 |
| MMRCO6-1 | 28 | 30 | 32 | 29 | 32 | 27 | 27 | 53 |
| F28B1 | 29 | 30 | 34 | 29 | 34 | 28 | 27 | 54 |
| M48-1B-1 | 29 | 30 | 36 | 29 | 34 | 30 | 29 | 55 |
| WE8B-23 | 30 | 33 | 35 | 31 | 35 | 29 | 29 | 56 |
| M67-1 | 31 | 21 | 1 | 32 | 24 | 1 | 22 | 57 |
| T12-1 | 32 | 35 | 37 | 33 | 36 | 31 | 6 | 58 |
| P2G | 32 | 43 | 7 | 7 | 36 | 38 | 34 | 59 |
| T18-1 | 33 | 36 | 29 | 34 | 18 | 32 | 30 | 60 |
| R11372 | 34 | 38 | 39 | 35 | 37 | 33 | 31 | 61 |
| R23087 | 35 | 41 | 41 | 37 | 39 | 36 | 33 | 62 |
| R23275 | 36 | 42 | 42 | 38 | 11 | 37 | 4 | 63 |
| P6N | 37 | 44 | 44 | 40 | 40 | 39 | 35 | 64 |
| Pn4D | 38 | 45 | 45 | 41 | 41 | 40 | 36 | 65 |
| CCUG 33920 | 39 | 46 | 46 | 42 | 42 | 41 | 37 | 66 |
| CCUG 34286 | 40 | 48 | 48 | 44 | 44 | 43 | 39 | 67 |
| MG1 | 41 | 49 | 50 | 46 | 45 | 44 | 40 | 68 |
| *A. naeslundii* |  |  |  |  |  |  |  |  |
| E1-20 | 42 | 50 | 51 | 47 | 46 | 45 | 41 | 69 |
| T17-3 | 42 | 50 | 56 | 54 | 51 | 45 | 46 | 70 |
| M42-1-1 | 42 | 50 | 56 | 54 | 51 | 45 | 46 | 70 |
| Pn16E | 42 | 50 | 64 | 58 | 51 | 45 | 46 | 71 |
| R24330 | 42 | 52 | 58 | 54 | 51 | 45 | 45 | 72 |
| T20P-1 | 42 | 54 | 56 | 53 | 52 | 45 | 44 | 73 |
| S44D | 42 | 59 | 61 | 57 | 56 | 54 | 47 | 74 |
| S49H | 42 | 60 | 58 | 58 | 51 | 45 | 45 | 75 |
| CCUG 34725 | 42 | 60 | 58 | 58 | 51 | 45 | 45 | 75 |
| R9841 | 42 | 61 | 62 | 53 | 51 | 45 | 46 | 76 |
| *A. naeslundii*^T^ (ATCC12104) | 42 | 61 | 64 | 54 | 51 | 45 | 44 | 77 |
| S65A | 42 | 62 | 62 | 54 | 51 | 45 | 46 | 78 |
| F12B1 | 43 | 51 | 52 | 48 | 47 | 46 | 42 | 79 |
| R19039 | 43 | 51 | 52 | 48 | 47 | 50 | 42 | 80 |
| S38H | 43 | 55 | 60 | 55 | 53 | 52 | 41 | 81 |
| S41C | 43 | 55 | 60 | 55 | 53 | 52 | 41 | 81 |
| U136-1 | 43 | 55 | 60 | 55 | 53 | 52 | 41 | 81 |
| R8152 | 43 | 57 | 63 | 56 | 58 | 57 | 48 | 82 |
| CCUG 37599 | 43 | 66 | 66 | 63 | 54 | 59 | 49 | 83 |
| F6E1 | 44 | 52 | 53 | 49 | 48 | 47 | 43 | 84 |
| G126D | 45 | 53 | 54 | 50 | 49 | 48 | 44 | 85 |
| Pn1GA | 45 | 55 | 60 | 55 | 53 | 52 | 45 | 86 |
| T14P-1 | 46 | 54 | 54 | 53 | 50 | 49 | 45 | 87 |
| G127B | 46 | 54 | 55 | 51 | 50 | 49 | 45 | 88 |
| G51C | 46 | 54 | 55 | 52 | 50 | 49 | 45 | 89 |
| T9P-1 | 46 | 56 | 58 | 51 | 50 | 46 | 46 | 90 |
| S53C | 46 | 61 | 55 | 59 | 57 | 55 | 44 | 91 |
| T23P-1 | 47 | 55 | 57 | 55 | 53 | 49 | 41 | 92 |
| MMRC12-1 | 48 | 52 | 53 | 60 | 53 | 47 | 43 | 93 |
| W8-2-3 | 48 | 57 | 59 | 56 | 54 | 50 | 42 | 94 |
| WE6B-3 | 48 | 57 | 59 | 56 | 47 | 51 | 42 | 95 |
| WE7B1 | 48 | 57 | 59 | 56 | 47 | 51 | 42 | 95 |
| S43L | 49 | 58 | 55 | 51 | 55 | 53 | 46 | 96 |
| MB-1 | 50 | 50 | 58 | 48 | 49 | 56 | 44 | 97 |
| R13240 | 51 | 63 | 59 | 54 | 47 | 58 | 42 | 98 |
| Pn6N | 52 | 64 | 65 | 61 | 54 | 59 | 43 | 99 |
| CCUG 35334 | 53 | 65 | 64 | 62 | 59 | 60 | 45 | 100 |

Strain 117 is *A. johnsonii* CCUG 33932=PK1259

*fimP* sequence 120 is from *A. viscosus* ATCC19246 [AF106034]
